# Improvement of Mixed-Mode I/II Fracture Toughness Modeling Prediction Performance by Using a Multi-Fidelity Surrogate Model Based on Fracture Criteria

**DOI:** 10.3390/ma15238580

**Published:** 2022-12-01

**Authors:** Attasit Wiangkham, Prasert Aengchuan, Rattanaporn Kasemsri, Auraluck Pichitkul, Suradet Tantrairatn, Atthaphon Ariyarit

**Affiliations:** 1 School of Manufacturing Engineering, Institute of Engineering, Suranaree University of Technology, Muang, Nakhon Ratchasima 30000, Thailand; 2 School of Civil Engineering, Institute of Engineering, Suranaree University of Technology, Muang, Nakhon Ratchasima 30000, Thailand; 3 School of Mechanical Engineering, Institute of Engineering, Suranaree University of Technology, Muang, Nakhon Ratchasima 30000, Thailand

**Keywords:** mixed-mode I/II, fracture criteria, artificial intelligence, Kriging, multi-fidelity surrogate model

## Abstract

Today, artificial intelligence plays a huge role in the mechanical engineering field for solving many complex problems and the problem with fracture mechanics is one of them. In fracture mechanics, artificial intelligence is used to predict crack behavior under various conditions such as mixed-mode loading. Many parameters are used for explaining the crack behavior under various conditions, but those parameters are obtained from destructive testing, in which usually, only one data point is obtained from each test. An artificial problem method requires a large amount of data to train the model to be able to learn crack behavior, which is a disadvantage of applying this method to fracture mechanics. To eliminate the disadvantage of the large amount of experiment data required for modeling, in this study, the small data obtained from the experiment along with data obtained from fracture criteria that were used for elementary prediction of mixed mode fracture toughness were used to create an artificial intelligence model. Data from the experiment was combined with fracture criteria data using the multi-fidelity surrogate model that is described in this study. The mixed mode I/II fracture toughness of the PMMA material was tested in order to primarily propose the data combination technique. After the modeling process, the prediction results indicated that the performance of a model in which the actual test data was combined with the data from the fracture criteria (multi-fidelity surrogate model) was more predictively effective compared to only actual data-based modeling.

## 1. Introduction

One of the crucial factors to consider when designing engineered components is fracture toughness. This parameter of the fracture mechanics field indicates a material’s capacity to withstand the crack expansion of discontinuous or cracked materials in the presence of external loads. According to the direction of the external load acting on the crack surface, fracture toughness can be divided into three modes [1]. For the majority of engineering parts, the load pattern or direction is more likely to occur in a mixed direction than in just one direction. According to the nature of the load direction on that part, it must be studied in a mixed-mode fracture toughness; either it is studied in mixed-mode I/II [2,3], mixed-mode I/III [4,5], or mixed-mode II/III [6,7], with mixed-mode I/II loading being popularly studied in various research. The fracture toughness values under mixed-mode loading can be calculated under several methods such as actual testing, numerical method, etc. In addition to such methods, in recent years, the artificial intelligence method was used to calculate or predict fracture toughness values.

Artificial Intelligence is one of the most effective solutions to complex problems based on human learning and decision-making. The majority of artificial intelligence models that predict fracture toughness use supervised learning which trains their models using data from actual fracture toughness tests in order to produce accurate predictions [8,9,10]. For this reason, the dataset used to train the model must be sufficiently large enough so that the model can learn the behavior of the material. The relatively data-intensive use of modeling artificial intelligence is considered a huge disadvantage in fracture toughness prediction. The fracture toughness test is destructive testing in which the test specimens are destroyed during testing and cannot be used for other testing anymore. According to the testing characteristics of fracture toughness, tests that are performed in several types of engineering materials such as polymers, metal, composites, or building materials usually obtain a single point of data from each test. As a result, gathering sufficient data to model artificial intelligence necessitates extensive actual testing, which appears to go against the idea that predictive modeling is used to minimize testing. In addition to the fracture toughness data from the actual test, when considering the method for determining the fracture toughness, a method by which the elementary fracture toughness can be calculated is known as fracture criteria, whereby a lower cost of data acquisition is compared to the actual test data.

The fracture criteria is a numerical model based on the relationship between stress, strain, or energy occurring around the crack tip of material. The fracture criteria was designed for pure model loading, but it can be extended to mixed-mode loading. Ordinarily, the fracture criteria is designed to indicate whether material is fractured or not by giving a specific value, but it can also be used as an elementary prediction of fracture toughness. Many fracture criteria are created based on the relationship around the crack tip. as Also of interest is the generalized maximum tangential stress criteria (GMTS) [11,12], which focuses on the tangential stresses at the crack tip, and the average strain energy density criteria (ASED) [13,14], which focuses on the strain energy density around the crack tip, and the generalized maximum energy released rate criteria (GMERR) [15], which concerns the strain energy release rate around the crack tip etc. The fracture criteria serves as an excellent indication of the fracture of the material, but sometimes the specific value of fracture toughness obtained from the fracture criteria is quite different from the actual fracture toughness, as seen in the previous work of Poapongsakorn et al. [5], which describes the time-dependence of epoxy resin that was found in the elementary fracture toughness from criteria clearly different from the actual fracture toughness value.

In order to solve the relatively data-intensive use of modeling artificial intelligence, as in the case of models where the fracture toughness of materials is applied and where the acquisition of data is limited due to high cost or limited material, or when it is difficult to prepare specimens such as composites materials that take a lot of time to be molded or metal materials that take a long time in the heat treatment process, etc., this research aims to create an artificial intelligence model using small data from actual fracture toughness tests. The data from the actual fracture toughness test are combined with the elementary prediction data obtained from the widely used fracture criteria, including the generalized maximum tangential stress criteria (GMTS), the average strain energy density criteria (ASED), and the generalized maximum energy released rate criteria (GMERR) in the model training stage to obtain enough data to train the model to be able to make accurate predictions. Poly (methyl methacrylate) or PMMA, which is frequently used in lab-scale experiments, was chosen to perform fracture toughness testing under mixed-mode I/II loading in order to concentrate on presenting an artificial intelligence modeling technique from a small actual test dataset by combining the data with fracture criteria. The incline crack specimen with three-point bend testing configurations was selected as described in the first section of this study. The widely used fracture criteria for elementary fracture toughness prediction are described in the second section. Combining actual testing data with elementary prediction data from fracture criteria for artificial intelligence modeling relies on the multi-fidelity surrogate model integration principle [16,17,18] commonly used for multiple data combinations in aircraft design and the Kriging algorithm [19,20,21], which is designed for modeling artificial intelligence from small datasets that are described in the third section. The performance of the artificial intelligence model generated from the data integration is compared with the performance of the model generated from the only actual test data shown in the last section of this study.

## 2. Mixed-Mode I/II Fracture Toughness

**Figure 1:**
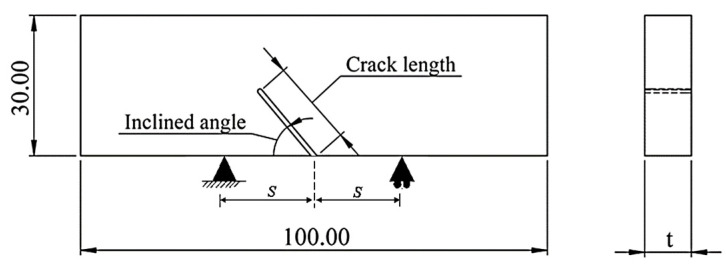
The dimension of ICB specimen.

This study aims to present a technique for improving artificial intelligence model prediction accuracy using a limited testing dataset. A poly (methyl methacrylate) or PMMA sheet, which is widely used in lab-scale testing, was employed to test the fracture toughness under mixed-mode I/II in order to concentrate largely on the presentation of the approach. Table 1 shows the mechanical properties of the PMMA sheet utilized in this study, for which testing was conducted in accordance with ASTM D638 and D732 standards. Numerous testing methods and specimens, such as incline edge crack asymmetric bending [3], compact tension shear [6], asymmetric four-point bending [22], semi-circular bending [23], Brazilian disc specimen [24], etc., are used to study mixed-mode I/II fracture toughness. In this study, the mixed-mode I/II of PMMA was studied using the inclined crack bending specimen based on previous research by Mingdong Wei [25] and M.R.M. Aliha [26]. The inclination crack bending specimen (ICB) was modified to be more suitable for our three-point bending testing apparatus by extending its width and length. The dimension of the ICB specimen is shown in Figure 1 (the crack length, inclined angle, span length (S), and thickness of specimen (t) will be explained in the next section).

**Figure 2:**
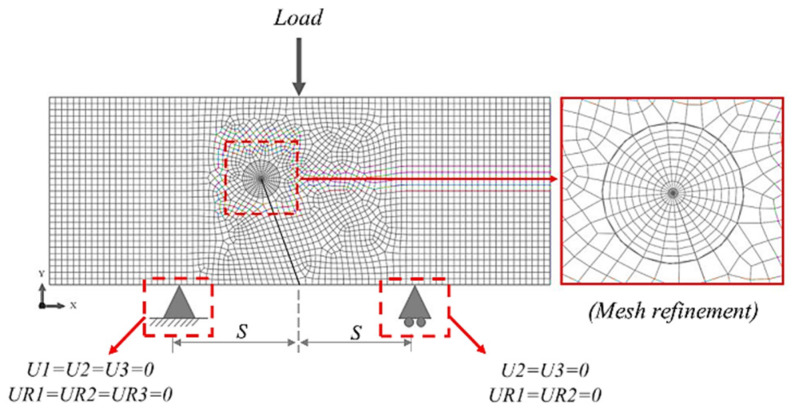
Finite element analysis model of the ICB specimen.

Stress intensity factors (K) were used to present the mixed-mode I/II fracture toughness values of PMMA in the form of the ICB specimen shape, which were divided into mode I ($K_{I}$) and mode II ($K_{II}$) stress intensity factors, which we will refer to as “fracture toughness” to avoid confusion. The $K_{I}$ and $K_{II}$ of the ICB specimen can be calculated using the Equations (1) and (2), respectively. For ICB specimens, the crack’s inclination at an angle to the line of load applied to the specimen causes a mixed load between the open crack loading (mode I) and the shear crack loading (mode II) to be created. The ratio between mode I and mode II loading, also called “mode mixity parameter ($M^{e}$)”, can be calculated using Equation (3)

(1) $$K_{I}=\frac{P}{Wt}\sqrt{\pi a_{0,ICB}}Y_{I}\left(\frac{a_{0,ICB}}{W},\frac{S}{W},\beta \right)$$

(2) $$K_{II}=\frac{P}{Wt}\sqrt{\pi a_{0,ICB}}Y_{II}\left(\frac{a_{0,ICB}}{W},\frac{S}{W},\beta \right)$$

(3) $$M^{e}=\frac{2}{\pi }\tan^{-1}\left(\frac{K_{I}}{K_{II}}\right)$$

where P is maximum load applied to the specimen, W is the specimen width, t is the specimen thickness, $a_{0,ICB}$ is the initial crack length, S is the span length that measures the distance from the center of the specimen to the bottom of the three-point bending pin in horizontal axis, β is the inclined crack angle, $Y_{I}$ and $Y_{II}$ are the normalized or dimensionless stress intensity factors for mode I and mode II, respectively, which depend on the specimen’s geometry and loading configurations. The corresponding values of $Y_{I}$ and $Y_{II}$ of the ICB specimen at different specimen geometries and loading configurations can be computed using the finite element analysis model (FEA). The FEA model of the ICB specimen in ABQUS 6.13 software is shown in Figure 2. The FEA modeling used material properties of PMMA (Table 1) and the load applied to the model was fixed at 1 kN. The FEA model of all specimen geometries and loading configurations was created by twenty nodes quadratic C3D20 elements (Hex-dominated) with a convergence at 1 mm, the approximate global element size. Mesh refinement was generated around the crack tip for singularity concerns of the stress-strain field around the crack tip (Figure 2 right-hand side) by the fifteen nodes quadratic C3D15 element (Wedge) with a 0.15 mm element size. For elements that are defined according to the above, the model had approximately 21,536 elements. According to actual testing, the moving condition of the FEA model was set, and the support pin on the bottom left side is set to be motionless or fixed to both move and rotate. The support pin on the bottom right side was configured as a roller support that can be moved along the x-axis and rotated in the direction normal to the x-y plane.

**Figure 3:**
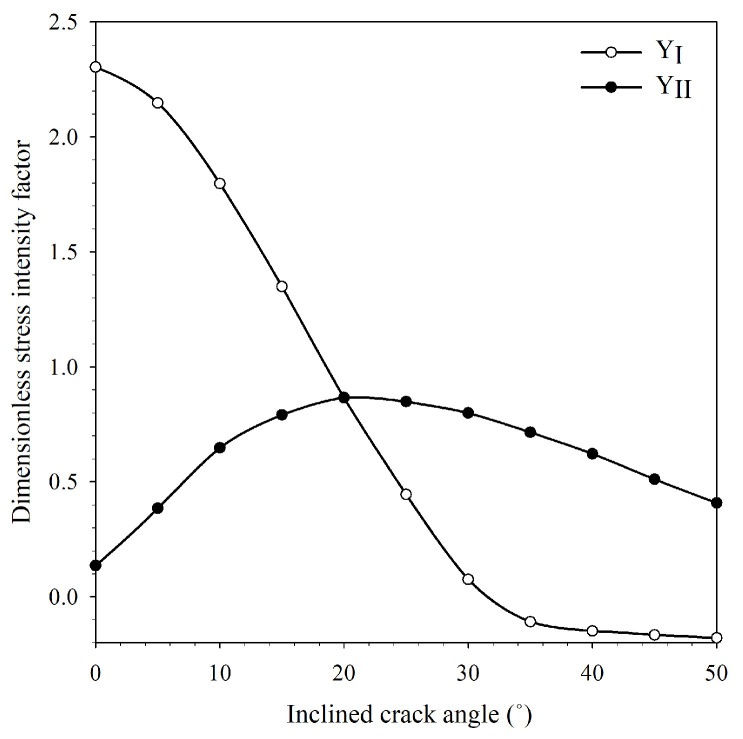
Mode I and II dimensionless stress intensity factor at various incline crack angles.

According to preliminary experiments, the length of the FEA model was set at 100 mm, the width at 30 mm, the ratio between initial crack length and specimen width set at 0.6, the ratio between span length and specimen width set at 0.6, and the incline crack angle (β) was increased from 0° to 50° (increment 5°). According to Equation (4), $Y_{I}$ and $Y_{II}$ can be converted from $K_{I}$ and $K_{II}$ extracted from the finite element analysis using the J-integral method [26,27]. The dimensionless stress intensity factor $Y_{I}$ and $Y_{II}$ at various incline crack angles are shown in Figure 3. The results showed that $Y_{I}$ decreased as the incline crack angle increased, whereas $Y_{II}$ increased until reaching the maximum value at an incline crack angle around 20° and then decreased. According to the FEA result, the incline crack angle was chosen to be 0° for pure mode I ($M^{e}$ = 1), 20° for mixed-mode I/II ($M^{e}$ = 0.5), and 31° for pure mode II ($M^{e}$ = 0). Fracture toughness testing was carried out on a Lloyd universal testing machine LD series (100 kN) at a loading rate of 10 mm/min. The ICB specimen was prepared using a laser cutting process on a PMMA sheet. A brand-new razor blade was used to cut the crack tip of the ICB specimens to ensure that it contained the theoretically sharp crack. In Equations (1) and (2), the mixed mode fracture toughness shown values were changed according to the function of stress (P/Wt) applied to the specimen. The stress function applied to the specimen is found to be related to the force exerted on the specimen (P) and the cross-sectional area of the stressed area (Wt). In this study, the thickness of the ICB specimen which influenced the cross-sectional area of the stressed area was included to factor in the design of the experiment (DOE). The general full factorial design was generated, with at least three replicates in each condition and the average fracture toughness of PMMA from testing used for the present predictions method is shown in Table 2.

(4) $$Y_{i}=\frac{K_{i}Wt}{P\sqrt{\pi a_{0,ICB}}},\;i=Mode\;I,II,III$$

## 3. Mixed-Mode I/II Fracture Toughness Prediction

### 3.1. Mixed-Mode I/II Fracture Criteria

For the branch of fracture mechanics that studies the behavior of cracks under various loading types, such as crack propagation, crack severity parameters, and so on, the occurrence behavior of cracks in a material can be explained by the relationship of stress, strain, or energy exerted on the crack of the material. As a result, many researchers attempted to predict crack behavior, such as whether fractures occur on materials or not when subjected to various loading conditions, using a variety of numerical equations based on a relationship of stress, strain, energy, or material behavior known as “fracture criteria”. A fracture criterion defines how fracture occurs on material by giving a specific value which could be used as an elementary fracture toughness predictive tool. Most fracture criteria are typically designed for pure mode loading conditions, but they have also been developed and applied to mixed-mode loading. In this study, the generalized maximum tangential stress (GMTS), the average strain energy density (ASED), and the generalized maximum energy release rate criteria (GMERR) were used for the elementary prediction of the mixed-mode I/II fracture toughness of PMMA. The brief fracture criteria used in this study is described in this section.

#### 3.1.1. Generalized Maximum Tangential Stress Criteria (GMTS)

**Figure 4:**
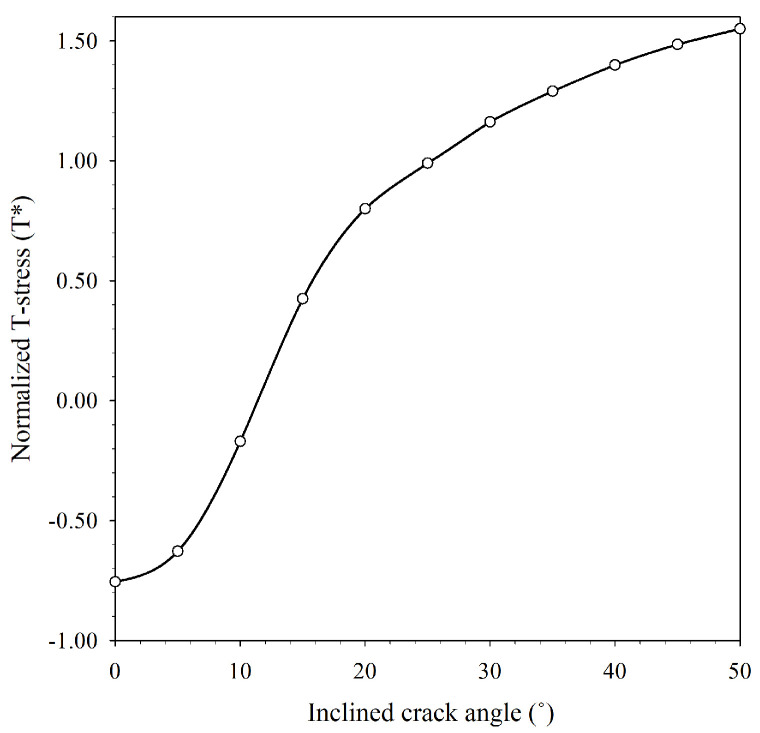
Normalized T-stress ($T^{*}$) of ICB specimen at a various inclined crack angles.

The generalized maximum tangential stress (GMTS) is a fracture criterion that was modified from maximum tangential stress (MTS) to improve the predictive performance of the fracture criteria. The GMTS criteria is a criterion based on the stress intensity factor (K) with added the T-stress term into the original MTS criteria. For the GMTS fracture criteria aimed at studying the tangential stresses at the crack tip ($\sigma_{\theta \theta }$), these stresses can be written according to the Equation (5) [25]

(5) $$\sigma_{\theta \theta }=\frac{1}{\sqrt{2\pi r}}\cos\frac{\theta_{0}}{2}\left(K_{I}\cos^{2}\frac{\theta_{0}}{2}-\frac{3}{2}K_{II}\sin\theta_{0}\right)+T\sin^{2}\theta_{0}+{\left(H.O.T\right)}_{\theta \theta }$$

where r is the distance to the crack tip in polar coordinates, $\theta_{0}$ is initial crack growth angle measured from the initial crack direction, T is a non-singular term or knows as T-Stress, and H.O.T. is the high order term in the stress solution. According to the GMTS fracture criteria, a material is fractured when the tangential stress at the crack tip exceeds the stress that the material can withstand, with the fracture growing in the direction of the maximum tangential stress at the crack tip. The direction of crack growth or initial crack growth angle ($\theta_{0}$) can be described in terms of dimensionless stress intensity factors that are the basis of the fracture criteria in Equation (6), where the estimated values of the initial crack growth angle ($\theta_{0}$) is equal to 0° at pure mode I ($M^{e}$ = 1), −52° at mixed-mode I/II ($M^{e}$ = 0.5), and −70° at pure mode II ($M^{e}$ = 0). The elementary prediction of mixed-mode I/II fracture toughness in the form of specific values of the GMTS criteria ($K_{I,GMTS}$ and $K_{II,GMTS}$) was obtained from the relationship of mode I critical fracture toughness ($K_{IC}$) described in Equations (7) and (8)

(6) $$\theta_{0}=-\cos^{-1}\left(\frac{3{\left(Y_{II}/Y_{I}\right)}^{2}+\sqrt{1+8{\left(Y_{II}/Y_{I}\right)}^{2}}}{1+9{\left(Y_{II}/Y_{I}\right)}^{2}}\right)$$

(7) $$\frac{K_{IC}}{K_{I,GMTS}}=\cos\frac{\theta_{0}}{2}\left[\cos^{2}\frac{\theta_{0}}{2}-\frac{3}{2}\frac{Y_{II}}{Y_{I}}\sin\theta_{0}\right]+\sqrt{\frac{2r_{C}}{a_{0,ICB}}}\frac{T^{*}}{Y_{I}}\sin^{2}\theta_{0}$$

(8) $$\frac{K_{IC}}{K_{II,GMTS}}=\cos\frac{\theta_{0}}{2}\left[\frac{Y_{I}}{Y_{II}}\cos^{2}\frac{\theta_{0}}{2}-\frac{3}{2}\sin\theta_{0}\right]+\sqrt{\frac{2r_{C}}{a_{0,ICB}}}\frac{T^{*}}{Y_{II}}\sin^{2}\theta_{0}$$

where $r_{C}$ is the critical distance (assume specimen failure under plane strain conditions), and, thus, $r_{C}$ can be estimated from Equation (9) [28]) and $T^{*}$ is the normalized form of T-stress, which is influenced by the same factors that influence dimensionless stress intensity factors. The T-stress can be calculated using the finite element analysis method and converted to a normalized form according to Equation (10). Figure 4 shows the $T^{*}$ at various incline crack angles. $T^{*}$ is a negative value at slightly inclined crack angles (less than 15°) and then increases with incline crack angles.

(9) $$r_{C}=\frac{1}{6\pi }{\left(\frac{K_{IC}}{\sigma_{t}}\right)}^{2}$$

(10) $$T=\frac{P}{Wt}T^{*}\left(\frac{a_{0,ICB}}{W},\frac{S}{W},\beta \right)$$

#### 3.1.2. Average Strain Energy Density Criteria (ASED)

The average strain energy density criteria (ASED) was first presented by Lazzarin et al. [29]. The ASED criteria focuses on strain energy density around the crack tip. In this criterion, the material will be fractured when an average strain energy density over a control volume reaches the critical strain energy ($W_{C}$), which is dependent on material properties and notch geometry. In the ICB, where the crack tip is sharp, the control volume is a circle of radius ($R_{C}$) with a center at the crack tip. The result of the stress-based expression can be summarized together with the fracture of the aforementioned material and can be rewritten in terms of mode I and mode II local strain density following Equation (11) [2]

(11) $$\frac{W_{1}}{W_{1C}}+\frac{W_{2}}{W_{2C}}=1$$

where $W_{1C}$ and $W_{2C}$ are the critical strain energy densities obtained from pure mode I and pure mode II loading, respectively, and can be calculated following Equations (12) and (13). $W_{1}$ and $W_{2}$ are the strain energy density generated from the applied external load. The values of $W_{1}$ and $W_{2}$ can be rewritten in terms of stress intensity factor, which is considered an elementary prediction of fracture toughness of the ASED criteria ($K_{I,ASED}$ and $K_{II,ASED}$) following Equations (14) and (15)

(12) $$W_{1C}=\frac{\sigma_{t}^{2}}{2E}$$

(13) $$W_{2C}=\frac{\tau_{t}^{2}}{2G}$$

(14) $$W_{1}=\frac{e_{1}}{E}\left[\frac{K_{I,ASED}^{2}}{R_{1C}{}^{2\left(1-\lambda_{1}\right)}}\right]$$

(15) $$W_{2}=\frac{e_{2}}{E}\left[\frac{K_{II,ASED}^{2}}{R_{2C}{}^{2\left(1-\lambda_{2}\right)}}\right]$$

where $e_{1}$ and $e_{2}$ are given by Lazzarin et al. [29] as 0.1186 and 0.3332, respectively, $K_{I,ASED}$ and $K_{II,ASED}$ are specific values of mixed-mode I/II fracture toughness obtained from the ASED criteria, $\lambda_{1}$ and $\lambda_{2}$ are mode I and II Williams’ eigenvalues, which are equal to 0.5 [30], and $R_{1C}$ and $R_{2C}$ are mode I and mode II radii under plane strain conditions, which can be calculated following Equations (16) and (17).

(16) $$R_{1C}=\frac{\left(1+v\right)\left(5-8v\right)}{4\pi }{\left(\frac{K_{IC}}{\sigma_{t}}\right)}^{2}$$

(17) $$R_{2C}=\frac{9-8v}{8\pi }{\left(\frac{K_{IIC}}{\tau_{t}}\right)}^{2}$$

where $K_{IIC}$ is mode II critical fracture toughness.

#### 3.1.3. Generalized Maximum Energy Release Rate Criteria (GMERR)

The generalized maximum energy released rate criteria was proposed by Hou, Cheng, et al. [15]. The GMERR was developed by adding terms of non-singular stress based on the maximum energy released rate criteria (MERR), which predicts the initiation of the mixed mode crack by calculating the energy release rate around the crack tip. In the GMERR criteria, materials will be fractured when values of strain energy release rate reach the maximum strain energy release rate of the material. The initial crack growth angle according to the GMERR can be calculated as follows

(18) $$C_{1}{\left(Y_{I}\right)}^{2}+C_{2}{\left(Y_{II}\right)}^{2}+C_{3}Y_{I}Y_{II}+4C_{4}\sqrt{\frac{r_{C}}{a_{0}}}Y_{I}T^{*}+4C_{5}\sqrt{\frac{r_{C}}{a_{0}}}Y_{II}T^{*}+16C_{6}\left(\frac{r_{C}}{a_{0}}\right){\left(T^{*}\right)}^{2}=0$$

where the variables are the same according to the Equation (7) and coefficients (C) can be calculated as follows

(19) $$\begin{array}{l} C_{1}=-\frac{1}{4}\sin\left(2\theta^{ER}\right)-\frac{1}{2}\sin\theta^{ER}\\ C_{2}=\frac{3}{4}\sin\left(2\theta^{ER}\right)-\frac{1}{2}\sin\theta^{ER}\\ C_{3}=-2\cos^{2}\theta^{ER}-\cos\theta^{ER}+1\\ C_{4}=10\sin^{5}\frac{\theta^{ER}}{2}-14\sin^{3}\frac{\theta^{ER}}{2}+4\sin\frac{\theta^{ER}}{2}\\ C_{5}=10\cos^{5}\frac{\theta^{ER}}{2}-20\cos^{3}\frac{\theta^{ER}}{2}+8\cos\frac{\theta^{ER}}{2}\\ C_{6}=\sin\left(2\theta^{ER}\right) \end{array}$$

The elementary prediction fracture toughness of the GMERR criteria separated by mode I and II loading ($K_{I,GMERR}$ and $K_{II,GMERR}$) can be expressed as

(20) $$\frac{K_{IC}}{K_{I,GMERR}}={\left[B_{1}+B_{2}{\left(\frac{Y_{II}}{Y_{I}}\right)}^{2}+B_{3}\frac{Y_{II}}{Y_{I}}+4B_{4}\sqrt{\frac{r_{C}}{a_{0}}}\frac{T^{*}}{Y_{I}}+4B_{5}\sqrt{\frac{r_{C}}{a_{0}}}\frac{Y_{II}T^{*}}{{\left(Y_{I}\right)}^{2}}+16B_{6}\frac{r_{C}}{a_{0}}\left(\frac{T^{*}}{Y_{I}}\right)\right]}^{\frac{1}{2}}$$

(21) $$\frac{K_{IC}}{K_{II,GMERR}}={\left[\begin{array}{l} B_{1}{\left(\frac{Y_{I}}{Y_{II}}\right)}^{2}+B_{2}+B_{3}\left(\frac{Y_{I}}{Y_{II}}\right)+4B_{4}\sqrt{\frac{r_{C}}{a_{0}}}\frac{Y_{I}T^{*}}{{\left(Y_{II}\right)}^{2}}+\\ 4B_{5}\sqrt{\frac{r_{C}}{a_{0}}}\frac{T^{*}}{Y_{II}}+16B_{6}\frac{r_{C}}{a_{0}}{\left(\frac{T^{*}}{Y_{II}}\right)}^{2} \end{array}\right]}^{\frac{1}{2}}$$

where coefficients (B) can be calculated as follows

(22) $$\begin{array}{l} B_{1}=\frac{1}{4}{\left(\cos\theta^{ER}+1\right)}^{2}\\ B_{2}=-3\sin^{4}\frac{\theta^{ER}}{2}+2\sin^{2}\frac{\theta^{ER}}{2}+1\\ B_{3}=-\frac{1}{2}\sin\left(2\theta^{ER}\right)-\sin\theta^{ER}\\ B_{4}=-4\cos^{5}\frac{\theta^{ER}}{2}+4\cos^{3}\frac{\theta^{ER}}{2}\\ B_{5}=4\sin^{5}{\frac{\theta }{2}}^{ER}-4\sin\frac{\theta^{ER}}{2}\\ B_{6}=\sin^{2}\theta^{ER} \end{array}$$

**Figure 5:**
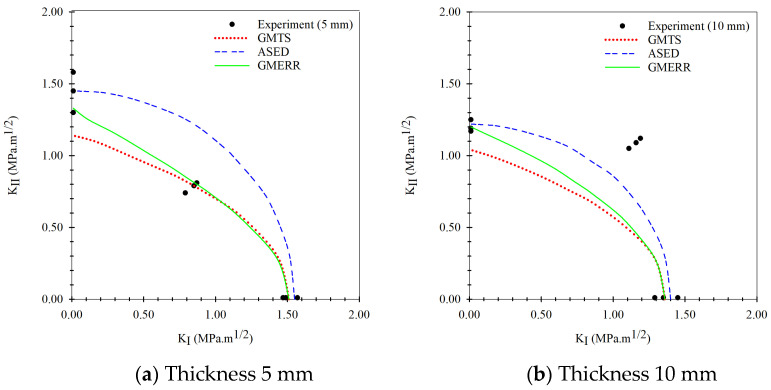
Elementary prediction of mixed-mode I/II fracture toughness of fracture criteria.

The fracture criteria mainly require the mechanical properties or critical mode I fracture toughness of materials for elementary prediction fracture toughness values that cause materials to fracture. In this study, the parameters used for the fracture criteria equations are shown in Table 1 and Table 2, and Figure 3 and Figure 4. The specific mixed-mode I/II fracture toughness from the criteria is calculated and separated based on the aforementioned influence of ICB specimen thickness on the cross-sectional area of the stressed area shown in Figure 5. The results showed good prediction performance at pure mode I and mode II fracture toughness for both thicknesses, but at mixed-mode I/II conditions, the predictions were quite different from the experimental results, indicating a decrease in the fracture criteria’s predictive efficiency. To demonstrate the fracture criteria’s predictive performance more clearly, the fracture criteria’s performance is measured using the MAPE performance metric, which is commonly used in regression problems according to Equation (23). The MAPE values for GMTS fracture toughness and experiment fracture toughness in modes I and II are 10.26% and 28.41 %, respectively. The MAPE values for ASED fracture toughness and experiment fracture toughness in modes I and II are 10.79% and 24.90%, respectively. The MAPE values for GMERR fracture toughness and experiment fracture toughness in modes I and II are 7.82% and 24.05%, respectively. Regarding the interpretation of MAPE values based on Lewis’ research [31], it was found that the predictive performance of the all fracture criteria at mode I ($K_{I}$) was relatively good (10% ≤ MAPE ≤ 20%). However, when mode II ($K_{I}{}_{I}$) is considered, prediction performance is a rational prediction, which means that only data trends (20% ≤ MAPE ≤ 50%) can be predicted. Although elementary predictions with fracture criteria show some deviation from actual fracture toughness, the specific fracture toughness from these fracture criteria is still used as a preliminary criterion that determines the limit stress that materials can withstand before fractures occur, which is very useful in designing engineering parts where fracture toughness is a consideration.

(23) $$MAPE=\frac{1}{n}\sum_{i=1}^{n}\left|\frac{A_{i}-\hat{A_{i}}}{A_{i}}\right|\times 100$$

where $A_{i}$ is the *i*th experiment data, $\hat{A_{i}}$ is the *ith* prediction data, and *n* is the number of observations.

### 3.2. Artificial Intelligence Method

In recent years, many researchers have applied artificial intelligence to many complex engineering problems in many disciplines. For fracture mechanics, artificial intelligence has been applied to describe the behavior of cracks, such as in the prediction of the pure mode loading fracture parameters [8,32,33] or mixed-mode loading fracture parameters [22,34]. Artificial intelligence methods have significant advantages in terms of making accurate predictions on complex problems, but there is a rather problematic disadvantage about the learning process of artificial intelligence models. The artificial intelligence learning process simulates how the human brain responds to external stimuli and learns to respond to that stimulus in the future, and learning in this manner requires a large amount of data. The fracture toughness test is a destructive test in which one data point is obtained, which means that applying artificial intelligence predictions requires a large number of tests, which appears to be counterproductive to the predictive equation’s goal of reducing the number of tests as much as possible.

In this study, the artificial intelligence model’s prediction performance is improved when data from the fracture toughness test is limited. The actual fracture toughness test data is then combined with the infinite elementary predictive data obtained from the fracture criteria. The concept of the data combination technique called the “multi-fidelity surrogate model” is described in the next section.

#### 3.2.1. Concept of Multi-Fidelity Surrogate Model

**Figure 6:**
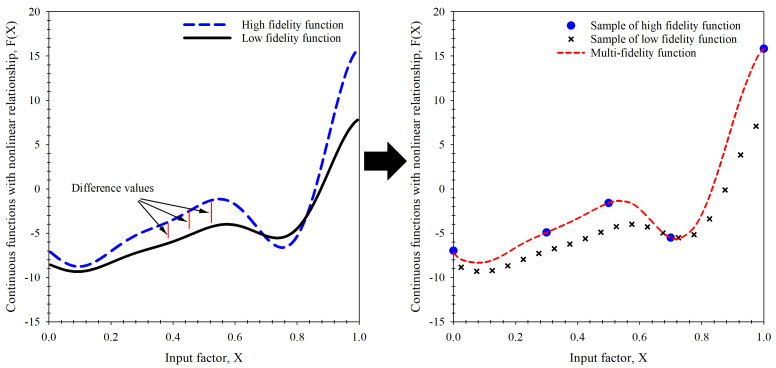
Concept of surrogate model for multi-fidelity function.

The concept of the combination technique is explained in Figure 6 using continuous functions with nonlinear relationships. (It should be noted that continuous functions with nonlinear relationships are unrelated to this study, as they are simply an illustration to help explain the concept of combination). The high fidelity function was used to represent the fracture toughness data obtained from actual testing. In addition, it has high accuracy but is expensive or time-consuming to obtain. The low fidelity function was used to represent the fracture toughness obtained from the elementary prediction of the fracture criteria where data acquisition is quick and inexpensive, even though accuracy may not be very high. The difference between the prediction results and the actual value of the problem is clearly shown in Figure 6 (left-hand side), but if the relationship between these two values is considered, the predicted value can be equal to the actual value of the problem according to Equation (24) when the difference is known. The actual value and prediction value difference parameters may be linear or nonlinear relationships, depending on the characteristics of the problem. This correlation occurs in such a way that the data is interchangeable and is performed on a model called the “surrogate model’. The interchangeability of the data means that rather than using an excessive amount of high fidelity data to build a model, low fidelity data can be used in conjunction with the difference parameters to build a predictive model of high fidelity data, as shown in Figure 6 (right-hand side). This model is referred to as a “multi-fidelity surrogate model”.

(24) $$F{\left(X\right)}_{HF,i}=F{\left(X\right)}_{LF,i}+\;D_{i}$$

where $F{\left(X\right)}_{HF,i}$ is the output of the high-fidelity function at index *i*th, $F{\left(X\right)}_{LF,i}$ is the output of the low-fidelity function at index *i*th, and $D_{i}$ is difference between the output of the high and low fidelity functions.

In the multi-fidelity surrogate model, data substitution is performed on the basis of a Gaussian process regression model known as the Kriging model. Although it uses a model to make the prediction, the Kriging model’s prediction does not directly predict the difference parameters but predicts that parameter along with the value of the high fidelity data or actual data. Prior to describing how the original Kriging model and the multi-fidelity surrogate model were implemented in the fracture toughness prediction, the preparation of the data and the performance evaluation of the artificial intelligence prediction model are described in the following section.

#### 3.2.2. Data Preparation and Model Performance Evaluation

Data preparation is one of the processes that affects the predictive performance of the model. In this study, the artificial intelligence model used specimen thickness (t) and the mode parameter ($M^{e}$) as inputs and average mixed-mode I/II fracture toughness ($K_{I}$ and $K_{II}$) as outputs. Six data points were used in the artificial intelligence modeling process. To reduce the different lengths of input factors in the artificial intelligence model (thickness and mixed mode parameter), input data was normalized to range 0 to 1 using Equation (25). Because of the small dataset of this study, the train and test dataset in the modeling process was selected by the holdout cross-validation method. All artificial intelligence models were created using MATLAB programing. For fracture toughness in which the data is continuous, the regression artificial intelligence model was used, in which many models’ performance metrics are evaluated. The performance of the prediction model was assessed in this study using the common regression performance metrics of mean absolute percent error (MAPE), root mean square error (RMSE), and coefficient of determination ($R^{2}$), which can be calculated in the following Equations (23), (26) and (27).

(25) $$X_{normalized}=\frac{X_{i}-X_{\min}}{X_{\max}-X_{\min}}$$

where $X_{i}$ is the input data of the artificial intelligence model at index *i*th, and $X_{\max}$ and $X_{\min}$ are the maximum and minimum data in the input dataset.

(26) $$RMSE=\sqrt{\frac{\sum_{i=1}^{n}{\left(A_{i}-\hat{A_{i}}\right)}^{2}}{n}}$$

(27) $$R^{2}=1-\left[\frac{\sum_{i=1}^{n}{\left(A_{i}-\hat{A_{i}}\right)}^{2}}{\sum_{i=1}^{n}{\left(A_{i}-\bar{A}\right)}^{2}}\right]$$

where $\bar{A}$ is the average of experimental data and other variables according to Equation (23).

#### 3.2.3. Brief of Kriging Model

**Figure 7:**
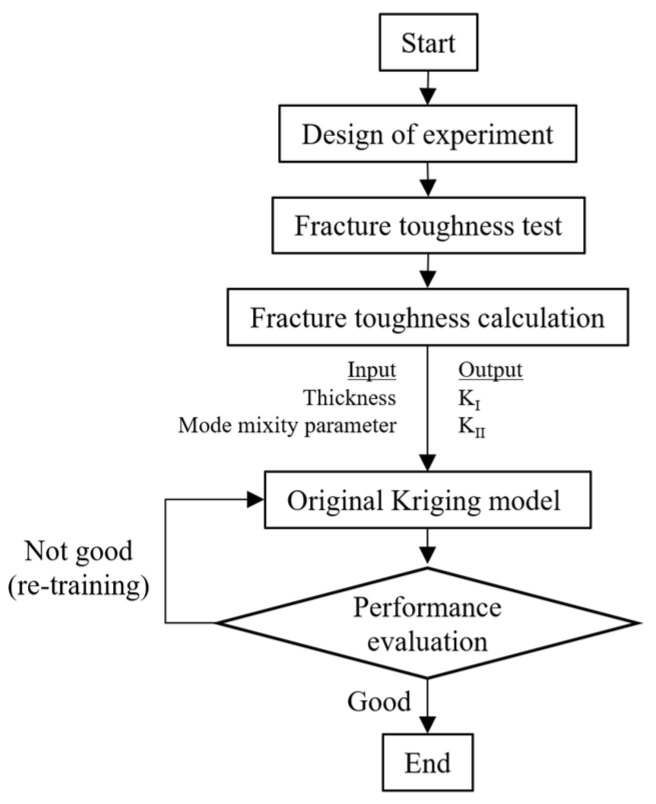
The modeling process of the original Kriging model.

The Kriging model or original Kriging model represents the single fidelity artificial intelligence model that used only experiment fracture toughness in the modeling process. In this section, the original Kriging abbreviation, also referred to as “Or-K”, was discussed. The Or-K modeling process is shown in Figure 7. The prediction model of the Or-K model can be expressed as follows [16,20].

The Or-K model can be predicting using the unknow function $\hat{y}\left(X\right)$ as

(28) $$\hat{y}\left(X\right)=u\left(X\right)+\varepsilon \left(X\right)$$

where u(X) denotes a global model and ε(X) denotes a local model. The sample of input X is interpolated using a Gaussian random function. The correlation between $Z\left(X_{i}\right)$ and $Z\left(X_{j}\right)$ is related to the distance between the two corresponding points $X_{i}$ and $X_{j}$. The distance function between point $X_{i}$ and $X_{j}$ is expressed as

(29) $$d\left(X_{i},X_{j}\right)=\sum_{k=1}^{n}\theta^{k}{\left|X_{i}^{k},X_{j}^{k}\right|}^{2}$$

where $\theta^{k}\left(0\leq \theta^{k}\leq \infty \right)$ is the kth element of the correlation vector parameter θ. The correlation between $X_{i}$ and $X_{j}$ is defined as

(30) $$\mathrm{Corr}\left[Z\left(X_{i}\right),Z\left(X_{j}\right)\right]=\exp\left[-d\left(X_{i},X_{j}\right)\right]$$

The Or-K model prediction can be expressed as

(31) $$\hat{y}(X)=u\left(X\right)+r^{T}R^{-1}\left(F-\hat{u}\right)$$

where $F={\left[f\left(X_{1}\right),f\left(X_{2}\right),f\left(X_{3}\right),\ldots ,f\left(X_{n}\right)\right]}^{T}$ is the output of evaluation function at $X=\left[X_{1},X_{2},X_{3},\ldots ,X_{n}\right]$. In this study, F represents the experiment fracture toughness obtained from the X input factors, R denotes the n×n matrix, whose (*i*, *j*) entry is $\mathrm{Corr}\left[Z\left(X_{i}\right),Z\left(X_{j}\right)\right]$, n is the number of observations sample, and r is the vector whose *i*th element is

(32) $$r_{i}\left(X\right)=\mathrm{Corr}\left[Z\left(X\right),Z\left(X^{i}\right)\right]$$

u(X) is assumed to be constant in the Or-K model, and $\hat{u}$ is given by

(33) $$\hat{u}=\left[u\left(X\right),u\left(X\right),u\left(X\right),\ldots ,u\left(X\right)\right]$$

It is defined as

(34) $$u\left(X\right)=\frac{I^{T}R^{-1}F}{I^{T}R^{-1}I}$$

where I denotes an *n*-dimensional unit vector.

The unknown parameter θ in Equation (29) is known as a hyperparameter that affects the prediction performance of the OR-K model, which can be estimated using the maximum likelihood estimation following

(35) $$\mathrm{Ln}\left(\mu ,\sigma^{2},\theta \right)=-\frac{n}{2}\ln\left(\sigma^{2}\right)-\frac{1}{2}\ln\left(\left|R\right|\right)$$

In this study, the generic algorithm optimization (GA) was used for the optimized maximum likelihood estimation. For given unknows of θ, the variance of Gaussian distribution ($\sigma^{2}$) can be defined as

(36) $$\sigma^{2}=\frac{{\left(F-\hat{\mu }\right)}^{T}R^{-1}\left(F-\hat{\mu }\right)}{n}$$

where the variables are the same according to the Equation (31).

#### 3.2.4. Brief of Multi-Fidelity Surrogate Model

**Figure 8:**
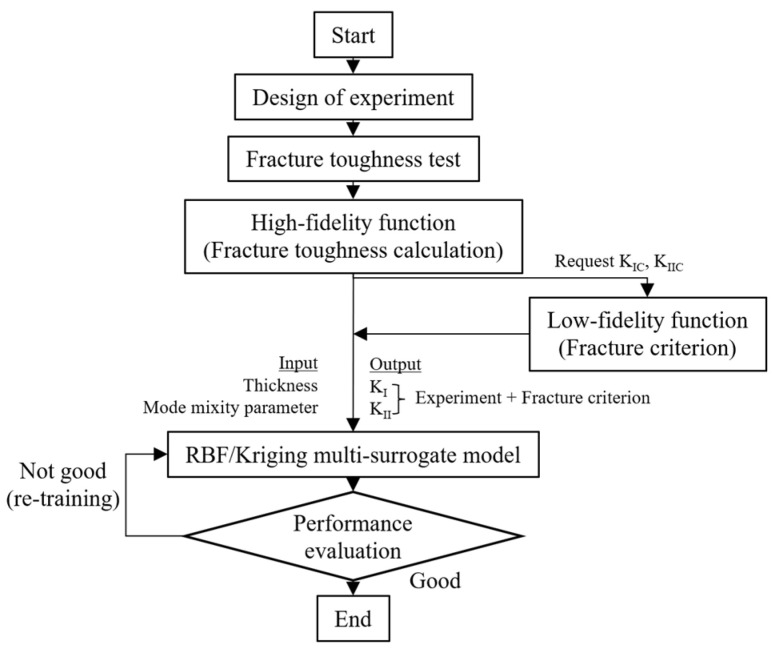
The modeling process of the multi-fidelity surrogate model.

As mentioned above, for the multi-fidelity surrogate model, data substitution is performed on the basis of the Kriging model. The multi-fidelity surrogate modeling process is shown in Figure 8. The surrogate model for multi-fidelity approaches a radius bias function (RBF) to represent the global model in Equation (37) (Note. High-fidelity data denotes fracture toughness data obtained from experiments and low-fidelity data denotes fracture toughness obtained from the fracture criteria)

(37) $$\hat{y}(X)=\left[\mu \left(X\right)+fr\left(X\right)\right]+r^{T}R^{-1}\left(F_{h}-\hat{\mu }-FR\right)$$

where local deviations $\left(r^{T}R^{-1}\left(F_{h}-\hat{\mu }-FR\right)\right)$ are evaluated based on the high fidelity data set obtained using the Or-K model $F_{h}={\left[f_{h}\left(X_{1}\right),f_{h}\left(X_{2}\right),f_{h}\left(X_{3}\right),\ldots ,f_{h}\left(X_{n}\right)\right]}^{T}$ and is the output data obtained from high-fidelity function at $X=\left[X_{1},X_{2},X_{3},\ldots ,X_{n}\right]$ and $FR={\left[f_{r}\left(X_{1}\right),f_{r}\left(X_{2}\right),f_{r}\left(X_{3}\right),\ldots ,f_{r}\left(X_{n}\right)\right]}^{T}$. Note that μ(X) is a mean value of the Gaussian process of the high-fidelity data, assumed to be a constant value expressed by Equation (34), and the definition of $\hat{\mu }$ is given by Equation (33). The term [μ(X)+fr(X)] is a compound of the Or-K model term of high fidelity data μ(X), which has been defined in Equation (28) and the RBF term of low fidelity data $f_{r}\left(X\right)$ predicted from the low fidelity data can be expressed as

(38) $$f_{r}\left(X\right)=a_{0}+a_{1}f_{l}\left(X\right)$$

where $f_{l}\left(X\right)$ is a function predicted by the RBF using low fidelity data, and $a_{0}$ and $a_{1}$ are correlation terms between the low fidelity and high fidelity data. The function predicted by the RBF using low fidelity data ($f_{l}\left(X\right)$) can be expressed as

(39) $$f_{l}\left(X\right)=\sum_{i=1}^{n}w_{i}\Phi \left(X-X_{i}\right)$$

where Φ(X) is an RBF, and $w_{i}$ is a weigh function. A multi-quadratic function is applied as an RBF. The weight is determined from the interpolation conditions

(40) $$Aw=F_{l}$$

where

(41) $$A=\left(\begin{array}{cccc} a_{1,1} & a_{1,2} & \cdots & a_{1,j}\\ a_{2,1} & a_{2,2} & \ldots & a_{2,j}\\ \vdots & \vdots & \ddots & \vdots \\ a_{i,1} & a_{1,1} & \ldots & a_{i,j} \end{array}\right)$$

where $a_{i,j}=\Phi \left(x_{i}-x_{j}\right)$ and $F_{l}={\left[f_{l}\left(X_{1}\right),f_{l}\left(X_{2}\right),f_{l}\left(X_{3}\right),\ldots ,f_{l}\left(X_{n}\right)\right]}^{T}$ are the values of the low fidelity function at $X=\left[X_{1},X_{2},X_{3},\ldots ,X_{n}\right]$.

The variance of Gaussian distribution ($\sigma^{2}$) can be defined as

(42) $$\sigma^{2}=\frac{{\left(F_{h}-\hat{\mu }-FR\right)}^{T}R^{-1}\left(F_{h}-\hat{\mu }-FR\right)}{n}$$

The unknown parameter (θ, $a_{0}$ and $a_{1}$) of multi fidelity-surrogated model can be estimated using the same maximum likelihood estimation as the Or-K model. According to the various type of low fidelity function (fracture criteria) that were used for the multi-fidelity surrogate model, the three multi-fidelity models were created. The model namely GM-K represents the multi-fidelity model based on the GMTS criteria, the AS-K model represents multi-fidelity model based on the ASED criteria, and the ER-K model represents the multi-fidelity model based on the GMERR criteria. For the multi-fidelity surrogate model, the data obtained from the experiment fracture toughness were used to create the model along with data obtained from the fracture criteria that was calculated by the separation of thee two thicknesses and mixed mode parameters from 0 to 1.0 with an increment 0.1 for each criterion.

## 4. Results and Discussions of the Artificial Intelligence Prediction Models

**Figure 9:**
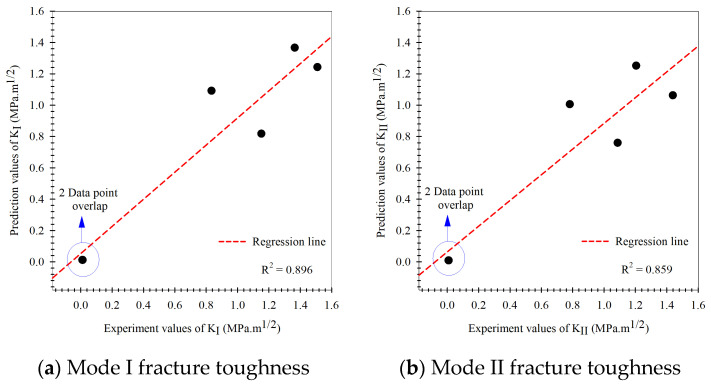
Prediction results of the original Kriging model compared to the experiment results.

**Figure 10:**
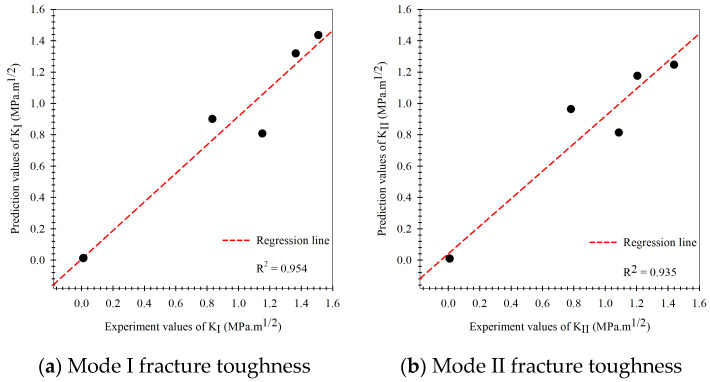
Prediction results of the multi-fidelity surrogate model based on the GMTS criteria compared to the experiment results.

**Figure 11:**
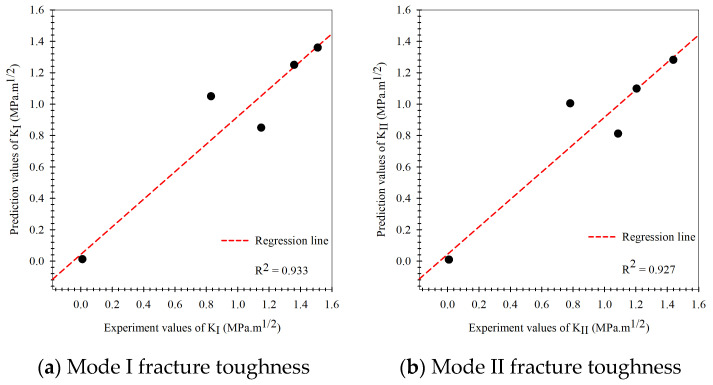
Prediction results of the multi-fidelity surrogate model based on the ASED criteria compared to the experiment results.

**Figure 12:**
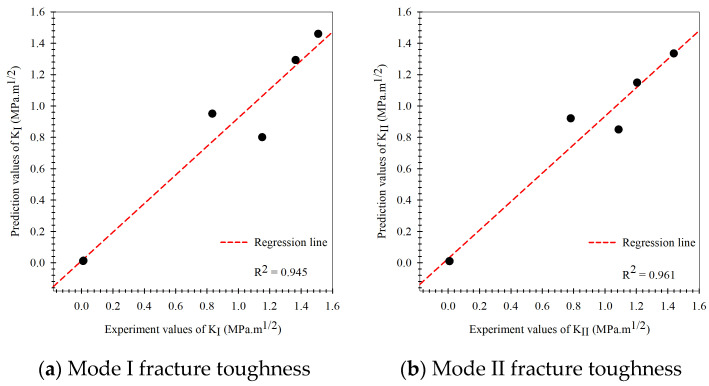
Prediction results of the multi-fidelity surrogate model based on the GMERR criteria compared to the experiment results.

According to the modeling process of the multi-fidelity surrogate model that combines high-fidelity data, a high precision response to the behavior of the output of the problems and low-fidelity data that show a low precision response to the behavior of the output of problems occur together in the modeling process. When closely considering this modeling method, also called the multiple certainties or multiple precision modeling methods, in which the high-fidelity data (actual experimental fracture toughness) represents the data with high certainty, whereas the low-fidelity data (fracture criteria) is simple to obtain but is inaccurate or has many errors, the data represents a low certainty response to the behavior of the output of the problems. The prediction results of the Or-K model in the modeling process are shown in Figure 9, which shows the modeling based on the experimental fracture toughness. To evaluate all artificial intelligence, the prediction performance metrics in different terms including the percentage-base error measurement (MAPE, Equation (23)), the scale based-error measurement (RMSE, Equation (26)), and the goodness-of-fit statistic ($R^{2}$, Equation (27)) between prediction results of the testing data set obtained via holdout cross-validation method and actual mixed mode I/II fracture toughness results were measured. When considering the prediction performance of the artificial intelligence model in mixed-mode I/II fracture toughness problem, the performances were measured separately into mode I and mode II according to the loading characteristic that causes specimens to fracture. The Or-K model had performance metrics including $R^{2}$, MAPE, and RMSE values equal to 0.896, 16.70%, 0.207 and 0.859, 14.79%, and 0.230 at mode I ($K_{I}$) and mode II ($K_{II}$) fracture toughness, respectively. The model performance metrics (MAPE) of the Or-K model demonstrate low prediction performance caused by a fundamental aspect of the artificial intelligence modeling due to the small dataset usage in modeling that affected the learning process (6 data points were used to generate the Or-K model). The prediction results of the multi-fidelity surrogate model, which aims to improve the prediction performance of the artificial intelligence mode in case the data used in the modeling process is limited, is shown in Figure 10, Figure 11 and Figure 12. The multi-fidelity surrogate model is based on the GMTS criteria (Figure 10), which used 28 data points in the modeling process (6 data from experiment and 22 data from criteria) and had $R^{2}$, MAPE, and RMSE values at mode I and II fracture toughness equal to 0.954, 12.41%, 0.147, and 0.935, 12.54%, and 0.156, respectively. The multi-fidelity surrogate model is based on the ASED criteria (Figure 11) and had $R^{2}$, MAPE, and RMSE values at mode I and II fracture toughness equal to 0.933, 15.07%, 0.170 and 0.927, 12.25%, and 0.164, respectively. The multi-fidelity surrogate model is based on the GNERR criteria (Figure 11) and had $R^{2}$, MAPE, and RMSE values at mode I and II fracture toughness equal to 0.945, 12.23%, 0.155 and 0.961, 12.28%, and 0.122, respectively. When the performance metrics of each multi-fidelity surrogate model were compared, it was found that firstly, for the $R^{2}$ values, the GM-K model had the highest mode I fracture toughness and the ER-K model had the highest at mode II fracture toughness. Secondly, for the MAPE values, the ER-K model had the lowest at mode I fracture toughness and the AS-K model had the lowest MAPE at mode II fracture toughness. Finally, for the RMSE values, the GM-K model had the lowest fracture toughness at mode I and the Ri-K model had the lowest fracture toughness at mode II.

The prediction performance metrics of the multi-fidelity surrogate model demonstrate that the accuracy of the model had increased when compared to the Or-K model, which only used data from experiments. The $R^{2}$ values of the highest accuracy model had improved when compared to the Or-K of around 6.47% and 11.87%, the MAPE values decrease around 26.77% and 17.17%, and the RMSE values decrease around 28.98% and 46.96% at mode I and II fracture toughness, respectively. When compared to the fracture criteria, unfortunately, the mode I fracture toughness from the multi-fidelity model shows a decrease in predictive performance. The MAPE values of the GM-K, AS-K, and ER-K models were increased by around 15.01%, 46.88%, and 56.39%, respectively, when compared to the based fracture criteria used for each modeling. The error of the multi-fidelity surrogate model at mode I fracture toughness occurs mainly in mixed-mode loading, consistent with the predicted results of the fracture criteria in mixed-mode loading (Figure 5) used as additional data in modeling, whereas under pure mode I and II loading, fewer errors occur compared with mixed-mode loading because prediction results of the fracture criteria are close to the experimental results because the fracture criteria requires fracture toughness under pure mode loading in the fracture criteria equation. It is for this reason that the multi-fidelity surrogate model has more errors than the fracture criteria; however, when considering the interpretation of the MAPE values, the performance of the three models was also considered to be a good predictor. In mode II fracture toughness, when compared to the fracture criteria, the multi-fidelity surrogated model efficiency increased. When considering the MAPE value, it was found that the MAPE values of the GM-K, AS-K, and ER-K models were decreased around 55.86%, 50.80%, and 48.94%, respectively. The decrease in MAPE showed a marked improvement in predictive performance, when before it was only possible to predict trends of the data when using the fracture criteria.

The additional fracture toughness was tested to measure the model prediction performance in datasets that are different from the modeling process. The prediction results of the multi-fidelity surrogated model with the same data and different levels of thickness and mode mix parameters are shown in Table 3 and Table 4 according to the mode loading. Further experimental results show that the fracture toughness obtained by the prediction model is very close to the experiment results. When considering mode I fracture toughness, the MAPE values of the Or-K model, GM-K model, AS-K model, and ER-K model are equal to 18.90%, 9.42%, 11.35%, and 8.90%, respectively, whereas at mode II, fracture toughness is equal to 9.83%, 3.33%, 3.33%, and 2.80%, respectively. The prediction results in Table 3 and Table 4 showed a very close prediction between the Or-K model and all multi-fidelity models in the levels of the input factors used to create the model. The prediction results model is very close because of the fact that the model had seen data at this level before in the modeling process. On the other hand, the prediction result of the Or-K model and all multi-fidelity models are quite different in the levels of the input factors that differ in the modeling process, where the multi-fidelity models have values that are clearly close to the experimental results. The above results show that modeling to predict mixed-mode I/II fracture toughness results from combining the experimental data with the fracture criteria to improve model accuracy in case of factor levels never seen before. This indicates that the multi-fidelity surrogated model can solve the prediction issue in the case of predicting fracture toughness of expensive or low-volume materials, making it impossible to perform a large number of tests.

## 5. Conclusions

This research aims to improve the prediction performance of artificial intelligence models (Kriging model) on mixed-mode I/II fracture toughness of PMMA, which is modeled using small datasets to respond in cases where data acquisition is limited. Prediction model improvement involves combining data obtained from experiment testing with data obtained from fracture criteria using a multi-fidelity surrogate model. The multi-fidelity surrogate model based on the fracture criteria includes the generalized maximum tangential stress criteria (GMTS), the average strain energy density criteria (ASED), and the generalized maximum energy released rate criteria (GMERR). The results of the research can be summarized as follows:

1. As for the fracture criteria, the elementary fracture toughness prediction results are very close to the experimental results under pure mode loading since the fracture criteria equations rely on critical fracture toughness under pure load ($K_{IC}$, $K_{IIC}$), which was obtained from the experiment. For the predicted results at mixed-mode loading, the values were found to be rather inconsistent with the experimental results.
2. As for the original Kriging model, the predicted fracture toughness was rather inaccurate compared to the experimental results in the modeling process. The model had $R^{2}$ values equal to 0.896 and 0.859, MAPE values equal to 16.70% and 14.79%, and the RMSE equal to 0.207 and 0.230 when considering modes I and II fracture toughness, respectively.
3. The prediction performance of the multi-fidelity surrogate model, which is modeled on experimental data as well as the elementary prediction data obtained from the fracture criteria, was found to be higher than that of the original Kriging model or the fracture criteria. For the multi-fidelity surrogate model, the model’s performance depends on the fracture criteria used in the modeling process.
4. The multi-fidelity surrogate model based on the GMTS criteria had $R^{2}$, MAPE, and RMSE equal to 0.954, 12.41%, 0.147, and 0.935, 12.54%, 0.156 following the mode I and II loading while model based on the ASED had R2, MAPE, and RMSE equal to 0.933, 15.07%, 0.170 and 0.927, 12.25%, 0.164 and the model based on the MERR had $R^{2}$, MAPE, and RMSE equal to 0.945, 12.23%, 0.155 and 0.961, 12.28%, 0.122, respectively.
5. The multi-fidelity surrogate model based on the fracture criteria will ostensibly perform better than the original Kriging model, which solely relied on experimental data in the modeling process, in case the input factors to be predicted differ from the input factors used in the modeling process.
6. The multi-fidelity models’ prediction performance indicated that they are very useful in situations where testing materials are difficult to obtain or prepare for in order to gather enough data to apply artificial intelligence techniques to the problem of fracture toughness. They also assist in reducing the costs associated with data acquisition.

## Figures and Tables

**Table 1:** Mechanical properties of PMMA used in this study.

Properties	Values
Tensile strength (MPa), $\sigma_{t}$	70.00 ± 3.67
Shear strength (MPa), $\tau_{t}$	43.00 ± 2.45
Young’s modulus (GPa), E	2.95 ± 0.78
Shear modulus (GPa), G	1.10 ± 0.14
Poisson’s ratio, v	0.30 ± 0.02

**Table 2:** Average mixed-mode I/II fracture toughness of PMMA.

Thickness (mm)	*M^e^*	*K_I_* (MPa·m^1/2^)		*K_II_* (MPa·m^1/2^)	
		Average	SD	Average	SD
5	1.0	1.509	0.052	0.009	0.002
5	0.5	0.854	0.041	0.825	0.039
5	0.0	0.012	0.001	1.439	0.110
10	1.0	1.365	0.080	0.008	0.002
10	0.5	1.152	0.039	1.107	0.036
10	0.0	0.010	0.001	1.205	0.042

**Table 3:** Mode I fracture toughness from models’ testing processes.

Factors		Experimental	Prediction Model			
*M^e^*	Thickness (mm)	*K_I_* (MPa·m^1/2^)	Or-K	GM-K	AS-K	ER-K
			*K_I_* (MPa·m^1/2^)	*K_I_* (MPa·m^1/2^)	*K_I_* (MPa·m^1/2^)	*K_I_* (MPa·m^1/2^)
0.5	5	0.887	0.834	0.845	0.844	0.844
0.3	7	0.993	0.851	0.981	0.989	0.999
0.8	8	0.321	0.210	0.361	0.372	0.355
0.5	9	1.175	1.151	1.160	1.181	1.165
0.0	10	0.010	0.015	0.007	0.006	0.007
0.5	10	1.081	1.151	1.155	1.151	1.151

**Table 4:** Mode II fracture toughness from models’ testing processes.

Factors		Experimental	Prediction Model			
*M^e^*	Thickness (mm)	*K_II_* (MPa·m^1/2^)	Or-K	GM-K	AS-K	ER-K
			*K_II_* (MPa·m^1/2^)	*K_II_* (MPa·m^1/2^)	*K_II_* (MPa·m^1/2^)	*K_II_* (MPa·m^1/2^)
0.5	5	0.854	0.782	0.785	0.782	0.784
0.3	7	0.657	0.789	0.631	0.682	0.647
0.8	8	1.253	0.989	1.204	1.292	1.206
0.5	9	1.168	1.087	1.190	1.201	1.185
0.0	10	1.190	1.204	1.208	1.205	1.209
0.5	10	1.073	1.087	1.080	1.079	1.070

## Data Availability

Not applicable.

## Nomenclature

**Symbol**	**Description**
$A_{i}$	Experiment data
$\bar{A}$	Average of experiment data
$\hat{A_{i}}$	Prediction data
ASED	Average strain energy density fracture criteria
$a_{0,ICB}$	Initial crack length
E	Young’s modulus
$F{\left(X\right)}_{HF}$	Output of high-fidelity function
$F{\left(X\right)}_{LF}$	Output of low-fidelity function
G	Shear modulus
GMTS	Generalized maximum tangential stress fracture criteria
GMERR	Generalized maximum energy release rate fracture criteria
H.O.T	High order term in the stress solution
ICB	Inclination crack bending specimen
$K_{IC}$	Critical mode I stress intensity factor
$K_{IIC}$	Critical mode II stress intensity factor
$K_{I}$, $K_{II}$	Mode I and mode II stress intensity factor
$K_{I,ASED}$,$K_{II,ASED}$	Mode I and mode I stress intensity factor from ASED criteria
$K_{I,GMTS}$,$K_{II,GMTS}$	Mode I and mode I stress intensity factor from GMTS criteria
$K_{I,GMERR}$, $K_{II,GMERR}$	Mode I and mode I stress intensity factor from GMERR criteria
$M^{e}$	Mode mix parameter
MAPE	Mean absolute percentage error
Or-K	Original Kriging model
P	Maximum load applied
$R^{2}$	Coefficient of determination
$R_{C}$	Radius of control volume
RMSE	Root means square error
r	Distance to the crack tip in polar coordinates
$r_{C}$	Critical distance
S	Span length of three-point bending
u(X)	Global model
T	T-Stress
$T^{*}$	Normalized form of T-stress
W	Specimen width
$W_{1}$, $W_{2}$	Mode I and mode II strain energy density
$W_{1C}$, $W_{2C}$	Mode I and mode II critical strain energy density
X	Input data of the artificial intelligence model
$Y_{I}$, $Y_{II}$	Mode I, II dimensionless stress intensity factor
β	Inclined crack angle
ε(X)	Local model
θ	Hyperparameter of Kriging model
$\theta_{0}$	Initial crack growth angle
$\lambda_{1}$, $\lambda_{2}$	Mode I and mode II Williams’ eigenvalues
v	Poisson’s ratio
$\sigma^{2}$	Gaussian distribution
$\sigma_{t}$	Tensile strength
$\sigma_{\theta \theta }$	Tangential stresses at the crack tip
$\tau_{t}$	Shear strength
(θ, $a_{0}$ and $a_{1}$)	Hyperparameter of multi-fidelity surrogate model
